# Awareness, attitude and behavior regarding proton pump inhibitor among medical staff in the Southwest of China

**DOI:** 10.1186/s12913-019-4725-6

**Published:** 2019-11-21

**Authors:** Hongli Luo, Qingze Fan, Tierong Bian, Xiuying Li, Kun Chen, Qingbi Zhang, Yuting Wei, Yang Xiao, Yan Li

**Affiliations:** 1 Department of Pharmacy, the Affiliated Hospital of Southwest Medical University, No. 25 Taiping Street, Jiangyang District, Luzhou, 646000 China; 2 Experimental Medicine Center,the Affiliated Hospital of Southwest Medical University, No. 25 Taiping Street, Jiangyang District, Luzhou, 646000 China; 3 School of Public Health, Southwest Medical University, No.1 Xianglin Street, Longmatan District, Luzhou, 646000 China

**Keywords:** Proton pump inhibitor, Awareness, Attitude, Behavior, Doctor, Nurse, Pharmacist

## Abstract

**Background:**

Proton pump inhibitors (PPIs) are one of the most frequently prescribed classes of drug in the world and there is a growing number of publications on correct versus incorrect use of PPIs worldwide. The knowledge of PPIs among the medical staff is essential for improving the rationality of PPI application. The present study aimed to investigate awareness, attitude and behavior toward PPI use among medical staff in the Southwest of China.

**Methods:**

The present descriptive-analytical study was conducted on 900 medical staff from three professional groups (300 doctors, 300 nurses and 300 pharmacists) in China. The study data were collected through a self-designed questionnaire which included demographics, awareness, attitude and behavior toward PPI use. The study was carried out in 22 hospitals in Luzhou between February and June 2018.

**Results:**

Of 900 surveys issued, 851valid questionnaires (295doctors, 268 nurses and 288 pharmacists) were returned. Of all respondents, 33.25% were men and 66.75% were women. The score related to PPI awareness score of medical staff was low (59.47 ± 15.75). The level of awareness of pharmacist was significantly higher than that of doctors and nurses (*P* < 0.01), which was related to gender, age, occupation, educational level, professional title, hospital nature and hospital grade. Similarly, on the attitude towards PPI use, the pharmacists scored also significantly higher than doctors and the nurses (*P* < 0.01). Three hundred eighty-one of 851 medical staff had used PPI in the past 1 year, of which omeprazole was the most widely used. Among doctors, nurses and pharmacists, the usage rate of PPI was 50.85, 42.16, 40.97%, respectively. The use frequency was related to occupation and professional title. The score about the behavior toward PPIs of the nurses was also significantly lower than that of doctors and pharmacists (*P* < 0.01).

**Conclusions:**

The study indicated that the medical staff lack of awareness concerning rational use of PPI in China, especially nurse. Thus, it is necessary to call for action on the improvement of PPI awareness and medication-taking behaviors to reduce PPI overuse and to promote the rationality of PPI application.

## Background

Proton pump inhibitors (PPIs) were proved to be currently the most effective drugs inhibiting hydrochloric acid secretion [1] and were widely used for the treatment and prophylaxis of upper gastrointestinal tract disorders including gastroesophageal reflux disease (GERD), peptic ulcer disease and its complications, Helicobacter pylori eradication therapy, dyspepsia, nonsteroidal anti-inflammatory drugs (NSAIDs)-induced ulcers, stress ulcers, and other hypersecretory conditions [2]. Since the first PPI omeprazole was introduced in 1989, other drugs in the class have been marketed: lansoprazole, pantoprazole, rabeprazole, esomeprazole and ilaprazole. There has been a substantial, continuing and unexplained rise in prescribing of PPIs. PPIs remain one of the world’s most frequently prescribed medications. In the United States, PPIs were prescribed in 4% of outpatients in 2002 and 9.2% in 2009, sales of PPIs accounted for about 10 billion dollars in 2007 and 13.9 billion dollars with 113 million prescriptions filled annually in 2010 [3]. In addition, PPIs have become the 8th therapeutic class on the prescription list in 2013. In Scotland, there was a threefold increase in PPI utilization between 2001 and 2017, however total expenditure on PPIs was 66.7% lower in 2017 compared to that in 2001 [4]. In the Netherlands, there was a threefold increase in PPI utilization between 2000 and 2010, whereas there imbursed expenditure fell by 58% in 2010, helped by increasing utilization of generic omeprazole at only 2% of the prepatent loss price in 2010 [5]. There was an eightfold increase in PPI utilization between 1997 and 2009, but only a twofold increase in reimbursed expenditure in Belgium [6]. Similar to other countries, there were considerable differences in the utilization of generics and patent-protected PPIs among Western European countries [7]. Several studies reported that the utilization and expenditure of PPIs had increased by 6 to 10 times over the past decade in several tertiary general hospitals in China [8, 9]. In China, although generic drugs are encouraged to use, their prices are only slightly lower than original drugs, which make the utilization and expenditure of PPI increase synchronously.

Overutilization is defined as prescribing PPIs without an appropriate indication or inappropriate continuation of PPIs upon discharge from the hospital. Unexplained abdominal pain was the main driver for prescribing intravenous PPIs empirically, out of which (68.9%) were for suspected upper gastrointestinal bleed [10]. The utilization of injectable PPIs increased substantially over the past decade at a tertiary hospital in China [8, 9]. A recent research showed that 86% of patients who were prescribed PPIs did not have appropriate indications in the general medical ward of a tertiary Jordanian hospital [2]. Another study showed that PPIs were taken by 25.4% of the patients hospitalized in an internal medicine department of a tertiary Greek hospital, but as many as 81.2% of them had no indications [11]. In our previous studies, all patients under going elective operations in hepatobiliary surgical were prescribed PPIs, but 82.41% of them had unlicensed indication. In addition, 35.59% of inpatients were prescribed PPIs, of which 57% had no indications [9, 12]. Furthermore, PPIs overuse in the clinical setting is often the result of incorrect stress ulcer prophylaxis (SUP) in non-intensive care unit patients, and failure to discontinue this therapy before hospital discharge, even in the absence of a therapeutic indication. Despite their perceived safety and reasonable costs, overutilization of PPIs would pose significant health risks such as gastrointestinal discomfort, dyspepsia, elevated liver transaminase, allergic reactions, visual abnormalities, osteoporosis, hypomagnesemia, community-acquired pneumonia, as well as *Clostridium difficile colitis* [4, 13].

Several postulated factors are responsible for inappropriate PPIs overutilization in China including misunderstandings about PPIs in medical staff (doctors, nurses and pharmacists), over prescribing of more expensive PPIs (injectable, original), tense physician-patient relationship and consumer-oriented advertising. Currently, in China, doctors prescribe prescriptions for patients, and then medication instruction is provided by nurses or pharmacists. Therefore, the knowledge and attitude towards PPIs in doctors, nurses and pharmacists can all affect the utilization and clinical efficacy of drugs. For example, previous studies indicated that clinical pharmacist’s real-time interventions facilitated the rational use of PPIs and resulted in favorable economic outcomes [12, 14]. If these medical staff do not fully understand the relevant knowledge of PPI, such as drug characteristics, pharmacological action, mechanism, indication, administration time, administration method, duration, drug interaction and adverse reaction, it is easy to cause overuse of PPIs, reduce efficacy and increase adverse reactions. But only a few studies focus on the awareness of PPI issues in medical staff. Therefore, given the current situation and possible causes of PPI overutilization in China, the purpose of this study was to investigate the awareness, attitude and behavior toward PPI use among doctors, nurses, and pharmacists in China so as to find some methods to improve the rationality of PPI application.

## Methods

### Questionnaire design

The present cross-sectional study was questionnaire-based and the questionnaire was designed on the basis of relevant guidelines for PPI applications and previous researches [15–17]. Then, the questionnaire was given to relevant experts in the field of gastroenterology, statistics and epidemiology to confirm its validity. Before the main survey, a small-scale pilot study was conducted in 50 medical staff. Based on the advice of experts and pilot study, the questionnaire was modified. The internal consistency of questions obtained in Cronbach’s alpha was 0.78. Hence, the reliability was confirmed. Consequently demographics and 33 revised questions were used in the questionnaire (Additional file 1). The first part of the questionnaire included demographic information of the respondents such as gender, age, education level, profession, professional title and nature, type and grade of hospital. The second, third, and fourth parts were, respectively, related to the level of awareness, attitude and behavior toward PPI use. The first 20 questions were related to the level of awareness. The answers for all the 20 questions were set up as ‘yes’ or ‘no’. Respondents selected ‘yes’ or ‘no’ based on their own knowledge and were encouraged to give only one answer to each question. Five or 0 points were assigned to the right or wrong answers, respectively. The next 6 questions, which were related to attitude, were regulated based on the five-point Likert scale with the scores being [18]: 5 for ‘completely agree’, 4 for ‘almost agree’, 3 for ‘indifferent’, 2 for ‘almost disagree’, and 1 for ‘completely disagree’. A higher score in first two categories represented better awareness of PPIs or more positive attitude. The last 7 questions were related to behavior toward PPI use. The first question was to investigate if the respondents had used PPIs. If respondents had used PPIs, he/she should answer the next 6 questions. The second question was the name of PPIs used. The other five-answer questions were graded with 1 point for always, 2 point for often, 3 for sometimes, 4 for seldom and 5 for never [17]. A higher score here presented a lower dependency on PPIs, corresponding to better PPIs usage behavior.

### Data collection

Respondents in this study were medical staff from 22 hospitals (ten grade-one hospitals, eight secondary hospitals and four tertiary hospitals) in Luzhou, China. We randomly selected 10, 50 and 100 respondents from each grade-one, secondary and tertiary hospital, respectively. The medical staff in the digestive department was excluded. We aimed to recruit at least 900 respondents (300 doctors, 300 nurses and 300 pharmacists). All respondents joined in with no incentives and were explained the aim of this survey. Before investigation, all members in the research team successfully completed a training program that tutored them to be familiar with the purpose of this study and methods. Data were collected by four research assistants from February to July 2018. This project was approved by the ethics committee of the Affiliated Hospital of Southwest Medical University. The research content and design of the project are in line with the ethical norms. Specifically, the four research assistants distributed anonymous questionnaire to each respondent and provided instructions after obtaining verbal informed consent to participate in the study. The instructions were as follows: ‘If you are willing to participate this survey, please complete this questionnaire independently and answer all the questions in a private area in approximately 10 minutes. You are not encouraged to check reference materials or consult others.’ The research assistants collected the questionnaires from respondents on the spot, checked the questionnaire for completion, and the respondents were encouraged to respond to any unanswered items. We predefined that a questionnaire was valid for analysis only if 100% of questions were answered.

### Statistical analysis

Collected data were entered into the Epidata 3.1 and SPSS19.0 statistical software and analyzed. For comparison between doctors, nurses and pharmacists, data were analyzed using chi-squared test or Wilcoxon rank sum test for categorical variables, and using one-way ANOVA for continuous variables. A *p*-value of less than 0.05 was considered statistically significant. Charts were performed using Graphpad Prism 6.0 software.

## Results

### Characteristics of respondents

A total of 900 questionnaires were distributed, out of which 851 were considered valid and suitable for analysis (response rate = 94.56%). Basic characteristics of respondents were provided in Table 1. The most populous group within the study were doctors—295 (34.67%) responders, followed by pharmacists —288 (33.84%) responders and nurses —268 (31.49%) responders. Two hundred eighty-three individuals (33.25%) were male and 568 individuals (66.75%) were female, since the nurse is still a mainly female domain in China. The average age of respondents was 36.42 ± 11.24. The majority of respondents worked in secondary or tertiary public comprehensive hospital.

**Table 1:** Characteristics of respondents [n(%)]

	Doctor (*n* = 295)	Nurse (*n* = 268)	Pharmacist (*n* = 288)	Total
Gender				
Male	145(49.15)	9(3.36)	129(44.79)	283(33.25)
Female	150(50.85)	259(96.64)	159(55.21)	568(66.75)
Age				
18~30	119(40.34)	95(35.45)	115(39.93)	329(38.66)
31~40	63(21.36)	44(16.42)	61(21.18)	168(19.74)
41~50	62(21.02)	103(38.43)	80(27.78)	245(28.79)
51~60	51(17.28)	26(9.70)	32(11.11)	109(12.81)
Education				
Senior high school	7(2.37)	50(18.66)	40(13.89)	97(11.40)
Associate degree	43(14.58)	87(32.46)	54(18.75)	184(21.62)
Baccalaureate	197(66.78)	125(46.64)	153(53.13)	475(55.82)
Master degree	36(12.20)	6(2.24)	38(13.19)	80(9.40)
Doctorate	12(4.07)	0(0.00)	3(1.04)	15(1.76)
Professional title				
Primary	135(45.76)	138(51.49)	143(49.65)	416(48.88)
Secondary	85(28.81)	108(40.30)	119(41.32)	312(36.66)
Senior	75(25.43)	22(8.21)	26(9.03)	123(14.46)
Hospital nature				
Public	275(93.22)	253(94.40)	275(95.49)	803(94.36)
Private	20(6.78)	15(5.60)	13(4.51)	48(5.64)
Hospital type				
Comprehensive	265(89.83)	228(85.07)	259(89.93)	752(88.37)
Specialized	30(10.17)	40(14.93)	29(10.07)	99(11.63)
Hospital grade				
Grade-one	27(9.15)	49(18.28)	17(5.90)	93(10.93)
Secondary	123(41.69)	136(50.75)	106(36.81)	365(42.89)
Tertiary	145(49.15)	83(30.97)	165(57.29)	393(46.18)

### The awareness of respondents regarding PPI knowledge

Table 2 presented the frequency of correct responses for each item related to awareness about PPI knowledge. Doctors, nurses and pharmacists responded with correct possibility higher than 50% in 13 (see Table 2 items 1,2,3,4,10,11,12,14,15,16,17,18,20) of 20 items. The top three highest rates of correct answers to questions was in the following items: ‘Do PPI cure acid-related diseases by inhibiting gastric acid secretion?’, ‘Do you think the more expensive or newer PPI will produce better and safer effect?’ and ‘Should PPI be swallowed as whole piece?’. On the other hand, only 12.46% of the respondents responded correctly to the duration of PPI prophylaxis. The frequency of correct responses for most questions in doctor and pharmacist was significantly higher than that in nurse, but there were no statistically significant difference for question 3, 11, 18 and 19 (*P* = 0.337, *P* = 0.444, *P* = 0.064 and *P* = 0.103). We expected that all medical staff would have high awareness scores, but in fact, they had an average score of only 59.47 ± 15.75 (Table 3). Compared with doctor group, the score of nurse was significantly lower (*P* < 0.01), but the score of pharmacist was significantly higher (*P* < 0.01). The score of PPI knowledge was related to gender, age, occupation, education level, professional title, hospital nature and grade (*P* < 0.05, Table 4).

**Table 2:** Frequency of correct responses about PPI knowledge in different medical groups [n (%)]

No	Questions (right answer)	Doctor (*n* = 295)	Nurse (*n* = 268)	Pharmacist (*n* = 288)	Total	χ^2^	*P*
1	Is PPI inactive prodrug? (yes)	202(68.47)	141(52.61)	215(74.65)	558(65.57)	31.559	0.000
2	Do PPIs include omeprazole, pantoprazole, lansoprazole, rabeprazole, esomeprazole, etc.? (yes)	226(76.61)	178(66.42)	224(77.78)	628(73.80)	11.114	0.004
3	Do PPI cure acid-related diseases by suppressing hydrochloric acid secretion? (yes)	275(93.22)	241(89.93)	266(92.36)	782(91.89)	2.175	0.337
4	Can PPI be used to prevent stress ulcer? (yes)	199(67.46)	156(58.21)	203(70.49)	558(65.57)	9.981	0.007
5	Can PPI be used to treat acute pancreatitis? (yes)	130(44.07)	88(32.84)	125(43.40)	343(40.31)	9.099	0.011
6	Does omeprazole have the largest individual difference compared with other PPIs? (yes)	131(44.41)	104(38.81)	152(52.78)	387(45.48)	11.137	0.004
7	Does omeprazole have the largest interaction compared with other PPIs? (no)	148(50.17)	99(36.94)	156(54.17)	403(47.36)	17.958	0.000
8	Does esomeprazole have the longest acid inhibition time compared with other PPIs? (yes)	140(47.46)	79(29.48)	159(55.21)	378(44.42)	38.917	0.000
9	Should omeprazole be selected for pediatric patients? (yes)	136(46.10)	92(34.33)	116(40.28)	344(40.42)	8.086	0.018
10	Should rabeprazole be selected for pregnant patients? (no)	183(62.03)	65(24.25)	223(77.43)	471(55.35)	167.007	0.000
11	Do you think the more expensive or newer PPI will produce better and safer effect? (no)	257(87.12)	226(84.33)	253(87.85)	736(86.49)	1.625	0.444
12	Is PPI usually available as enteric-coated capsules or tablets? (yes)	234(79.32)	185(69.03)	227(78.82)	646(75.91)	10.149	0.006
13	Should PPI usually be taken at breakfast? (yes)	133(45.08)	42(15.67)	244(84.72)	419(49.24)	267.932	0.000
14	Should PPI be taken after meal? (no)	198(67.12)	151(56.34)	242(84.03)	591(69.45)	51.299	0.000
15	Should PPI be swallowed as whole piece? (yes)	253(85.76)	205(76.49)	260(90.28)	718(84.37)	20.669	0.000
16	Is it advisable to increase thedose frequency rather than a single dose to improve effect? (yes)	174(58.98)	94(35.07)	155(53.82)	423(49.71)	35.055	0.000
17	Should patients take PPI for only 7 days in the Helicobacter pylori eradication therapy? (no)	215(72.88)	159(59.33)	204(70.83)	578(67.92)	13.534	0.001
18	DoesPPI treatment of gastric ulcer take 2 weeks to 4 weeks? (no)	168(56.95)	127(47.39)	157(54.51)	452(53.11)	5.497	0.064
19	Is duration of PPI prophylaxis until no high risk factors, or able to tolerate enteral feeding? (yes)	40(13.56)	24(8.96)	42(14.58)	106(12.46)	4.537	0.103
20	Do you think long-term use of PPI may cause adverse reactions such as osteoporosis, pneumonia, etc.? (yes)	202(68.47)	179(66.79)	219(76.04)	600(70.51)	6.608	0.037

**Table 3:** The average score of awareness, attitude and behavior on PPI use

	Doctor	Nurse	Pharmacist	Total
Awareness	61.78 ± 12.90^#^	49.14 ± 14.40^*^	66.70 ± 14.59^*#^	59.47 ± 15.75
Attitude	22.14 ± 4.10	21.91 ± 3.69	23.93 ± 3.49^*#^	22.68 ± 3.88
Behavior	20.68 ± 2.25^#^	18.35 ± 2.65^*^	20.93 ± 2.50^#^	20.04 ± 2.72

Compared with doctor group, ^*^P < 0.01; Compared with nurse group, ^#^*P* < 0.01

**Table 4:** The score of awareness about PPI knowledge and its influencing factors

	Score	*F*	*P*
Gender		12.713	0.000
Male	62.17 ± 14.28		
Female	58.12 ± 16.27		
Age		6.633	0.000
18~30	57.80 ± 15.23		
31~40	64.14 ± 16.62		
41~50	58.45 ± 15.47		
51~60	59.59 ± 15.28		
Occupation		115.849	0.000
Doctor	61.78 ± 12.90		
Nurse	49.14 ± 14.40		
Pharmacist	66.70 ± 14.59		
Education		9.065	0.000
Senior high school	56.91 ± 13.45		
Associate degree	56.93 ± 16.94		
Baccalaureate	59.34 ± 15.36		
Master degree	68.63 ± 14.65		
Doctorate	62.33 ± 15.45		
Professional title		3.067	0.047
Primary	58.33 ± 14.67		
Secondary	59.90 ± 16.61		
Senior	62.20 ± 16.72		
Hospital nature		3.916	0.048
Public	59.73 ± 15.74		
Private	55.10 ± 15.35		
Hospital type		0.007	0.935
Comprehensive	59.48 ± 15.83		
Specialized	59.34 ± 15.19		
Hospital grade		4.453	0.012
Grade-one	56.45 ± 15.56		
Secondary	58.51 ± 15.47		
Tertiary	61.07 ± 15.90		

### The attitude of respondents regarding PPI use

Table 3 showed that on the questions of attitude on PPI use, the pharmacists scored significantly higher than doctors or nurses (*P* < 0.01). Through analysis of responses to each question, it was found that the majority of respondents believed that overuse of PPI was common at present in China, and the main cause of PPI overuse was doctors’ or patients’ abuse of PPI.53.47% of pharmacists thought that the main purpose of PPI overuse was SUP, and more pharmacists held this opinion than doctors (45.08%) or nurses (25.37%) (*P* < 0.001). Furthermore, 77.78% of pharmacists considered that overuse of PPI would cause an increase in adverse drug reaction and medical cost, while only 66.78% of doctors and 70.15% of nurse did so (*P* = 0.011). In addition, 72.03% of respondents thought it was necessary to launch certain large scale education for medical staff and the public to promote better understanding about PPI (Table 5).

**Table 5:** Respondents’attitude on usage of PPI [n(%)]

Questions (completely agree, almost agree)	Doctor (*n* = 295)	Nurse (*n* = 268)	Pharmacist (*n* = 288)	Total	χ^2^	*P*
Overuse of PPI is common at presentin China.	258(87.46)	220(82.09)	254(90.97)	732(86.02)	5.081	0.079
The main cause of PPI overuse is doctors’ or patients’ abuse of PPI.	190(64.41)	192(71.64)	208(72.22)	590(69.33)	5.169	0.075
The main purpose of PPI overuse is SUP.	133(45.08)	68(25.37)	154(53.47)	355(41.72)	47.188	0.000
Overuse of PPI will cause an increase in adverse drug reaction and medical cost.	197(66.78)	188(70.15)	224(77.78)	609(71.56)	9.046	0.011
Necessary to carry out large scale education on rational use of PPI for medical staff and the public.	208(70.51)	172(64.18)	233(80.90)	613(72.03)	19.793	0.000
Necessary to strengthen the management of community pharmacy.	124(42.03)	84(31.34)	107(37.15)	315(37.02)	6.888	0.032

### The behavior of respondents toward PPI use

**Fig. 1:**
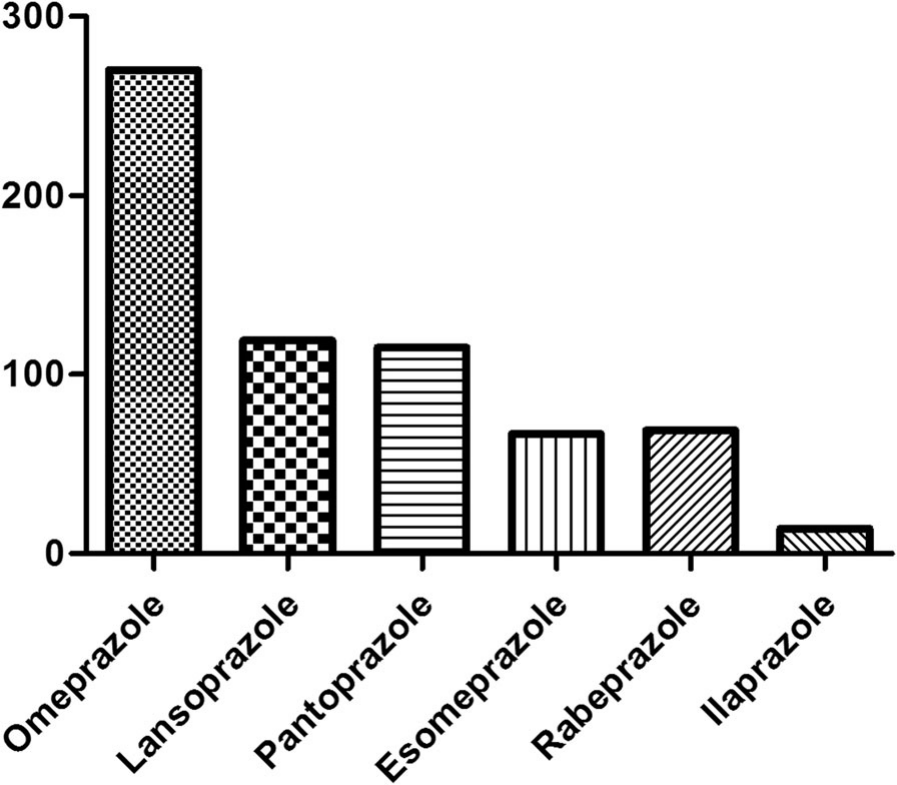
The number of respondents who have used PPI in the past 1 year

Three hundred eighty-one (150 doctors, 113 nurses, 118 pharmacists) of 851 respondents used PPIs in the past 1 year and omeprazole was the most widely used (Fig. 1). Nearly half of these respondents used two or more than two kinds of PPI. Among doctors, nurses and pharmacists, the usage rate of PPI was 50.85, 42.16, 40.97%, respectively. The use frequency was related to occupation and professional title (Table 6).

**Table 6:** The usage of PPIand its influencing factors

	Used	Unused	usage rate(%)	χ^2^/Z	*P*
Gender				0.601	0.438
Male	132	151	46.64		
Female	249	319	43.84		
Age				0.554	0.580
18~30	134	195	40.73		
31~40	93	75	55.36		
41~50	108	137	44.08		
51~60	46	63	42.20		
Occupation				6.823	0.033
Doctor	150	145	50.85		
Nurse	113	155	42.16		
Pharmacist	118	170	40.97		
Education				0.127	0.899
Senior high school	45	52	46.39		
Associate degree	82	102	44.57		
Baccalaureate	208	267	43.79		
Master degree	38	42	47.50		
Doctorate	8	7	53.33		
Professional title				2.395	0.017
Primary	169	247	40.63		
Secondary	150	162	48.08		
Senior	62	61	50.41		
Hospital nature				0.563	0.453
Public	357	446	44.46		
Private	24	24	50.00		
Hospital type				0.13	0.719
Comprehensive	335	417	44.55		
Specialized	46	53	46.46		
Hospital grade				1.596	0.111
Grade-one	51	42	54.84		
Secondary	162	203	44.38		
Tertiary	168	225	42.75		

It is interesting to note that our data revealed that nurse scored lower than the doctor and the pharmacist on the behavior towards the use of PPIs (*P* < 0.01, Table 3). When symptoms of stomachache, abdominal distension, nausea, vomiting or sour regurgitation occurred, the nurse would advocate the use of PPI more frequently than the doctor or the pharmacist (Table 7).

**Table 7:** Respondents’ behavior of using PPI

Questions (always, often)	Doctor (*n* = 295)	Nurse (*n* = 268)	Pharmacist (*n* = 288)	Total	χ^2^	*P*
Use PPI when abdominal pain	13(4.41)	45(16.79)	21(7.29)	79(9.28)	27.625	0.000
Use PPI when ventosity	24(8.14)	47(17.54)	36(12.50)	107(12.57)	11.294	0.004
Use PPI when nausea	12(4.07)	22(8.21)	20(6.94)	54(6.35)	4.315	0.116
Use PPI when vomiting	20(6.78)	41(15.30)	29(10.07)	90(10.58)	10.894	0.004
Use PPI when acid reflux	30(10.17)	50(18.66)	42(14.58)	104(14.34)	8.258	0.016

## Discussion

Knowledge about PPI therapy among the residents or medical staff of many countries including China has only been studied to a limited extent. This study surveyed medical staff from more than 20 hospitals in the Southwest of China to assess their awareness, attitude and behavior regarding PPIs in the form of questionnaires.

The result of awareness studies indicated that medical staff did not have good overall PPI knowledge. In particular, nurses got less information about PPIs than doctors or pharmacists, especially in aspects of physical and chemical properties, action mechanism, time and method of taking medicine and the characteristics of various PPI. For example, a randomized research have shown that inhibition hours (on 24hintragastric pH > 4.0) of esomeprazole 40 mg, rabeprazole 20 mg, omeprazole 20 mg, lansoprazole 30 mg and pantoprazole 40 mg inpatients with heartburn symptoms was 14.02, 12.13, 11.08, 11.52, 10.07, respectively [19]. While, in this study, only 29.48% of nurses knew esomeprazole has the longest acid inhibition time. Prescribers should take into account that use of PPI can cause toxicity risks, which remain to be fully characterized in pregnancy and children. PPIs, except omeprazole, can be given considering the benefit-harm ratio for the mother and fetus after the first trimester [20]. Recently researcher reviewed the reported adverse effects of PPIs (omeprazole, lansoprazole, pantoprazole, rabeprazole, esomeprazole) used in the treatment of pediatric GERD and found the rate of at least one adverse effect was 34, 43.7, 40, 61.5, 34.8%, respectively [21]. Therefore, omeprazole is recommended in pregnancy and children. Our survey indicated that less than half of the medical staff mastered this knowledge. Several clinical practice guidelines recommend that SUP should be discontinued until no high risk factors, or able to tolerate enteral feeding, or not receiving mechanical ventilation or not in ICU [15, 22]. However, we found that only 12.46% of the respondents responded correctly to the duration of PPI prophylaxis in present study. The score of PPI knowledge was related to gender, age, occupation, education level, professional title, hospital nature and grade. In China, the nurse professional is still a mainly female domain, and the education level and the professional title of nurses are lower than those of doctors and pharmacists. Moreover, hospital nature and grade can also affect the awareness about PPI knowledge. Medical staff in public hospitals got high score than staff in private hospitals. This is probably due to the fact that private hospitals usually put more focus on economic benefits, whereas public hospitals pay more attention to the professional training of their medical staff [23]. So, in public hospitals, medical staff has more opportunity of getting clinical practice experience and new knowledge, which will lead to more abundant knowledge about the reasonable use of PPIs.

There is increasing evidence supporting the overuse of PPIs in clinical practice in the world. More recently, researches have uncovered similar phenomena regarding PPI overuse in China [9, 24, 25]. Our previous study showed that 35.59% of inpatients were prescribed PPIs, of which 57% had no indications. Two other studies also found that the majority of surgical patients (59.26 and 67.03%, respectively) had received PPIs for SUP without indications and for long durations. This overutilization of PPIs for SUP in hospital can cause large economic waste and side effects such as gastrointestinal discomfort, dyspepsia, allergic reactions and visual abnormalities [4, 13]. Fortunately, in the present survey, the majority of respondents were aware of the prevalence of PPI overuse in China. Furthermore, respondents thought overuse of PPI was closely related to the misuse of the PPI by doctors or patients, and it was necessary to launch certain large scale education on rational use of PPI for medical staff to promote better understanding on PPI. Besides using PPIs for SUP in hospital, it is common to use PPIs for community long-term therapy, such as treating GERD. In China, oral omeprazole is the only over-the-counter (OTC) PPIs medicine. However, all oral PPIs, whether OTC or not, are available at community pharmacies without doctor’s prescription and the purchase quantity is not limited. Owing to this easy access to PPIs and poor knowledge of patients, there also remains overutilization of PPIs in community pharmacy. Some research have found that inappropriate long term use of PPIs can increase side effects such as community-acquired pneumonia, fragility fractures, hypomagnesemia, and *Clostridium difficile* infections [4]. In addition, except ilaprazole, the other five available PPIs (omeprazole, lansoprazole, pantoprazole, rabeprazole and esomeprazole) are all listed in the national medical insurancecatalogue. If a patient buys PPIs in a hospital, the cost can be co-payment. However, if a patient purchases PPIs at a community pharmacy, he will have to pay all by himself. Consequently, the overutilization in community pharmacy can increase patients’ medical burden. As a result, it is imperative to strengthen the management of community pharmacy and implement education on rational use of PPIs for community pharmacists and for the public. Unfortunately, only 37.02% of medical staff paid close attention to the community pharmacy management.

Nearly half of the respondents used PPIs in the past 1 year. In addition, nearly half of respondents used two or more than two kinds of PPIs. Compared to other PPIs, omeprazole was more frequently used, which was mainly related to the following reasons: First, omeprazole is the only OTC drug and consumer-oriented advertising about effectiveness and safety of omeprazole is easily acquired. Second, omeprazole is first introduced and applied clinically for the longest time, with the highest clinical evidence. Third, among all the six available PPIs, omeprazole, is the only one which is listed in the catalogue of national essential medicine in China.

Despite the useful information learnt from this study, several limitations require mention. First, our findings about knowledge, attitude and behavior regarding PPI among medical staff are based on self-reported instruments, which may introduce reporting bias and overestimation of positive knowledge, attitude and behavior. Second, because this was a survey from one geographic location in the Southwest of China, generalization of the results from these data may be limited. Another limitation of this study was the use of a self-designed questionnaire. Therefore, this scoring system may have some defects. In order to reduce defects, the questionnaire was designed and modified based on the advice of experts and pilot study. Finally, the number of respondents might be a little bit small owing to the shortage of research funding.

## Conclusion

The present study indicated that medical staff did not have satisfied PPIs awareness, attitude and behavior in more than 20 hospitals in the Southwest of China, especially nurses. This survey also truly reflected some of the potential causes of PPI abuse in the typical Chinese hospitals. To promote awareness of medical staff and to avoid PPI misuse, the authors suggest a number of fundamental keystones as followings: (1) Carrying out periodic training about PPI rational use for medical personnel. (2) Using all media means to establish educational programs about the illness that require PPI therapy and to emphasize when PPI will not do any good. (3) Emphasizing pharmacists’ role and responsibility in stopping PPI sale without prescription, except for OTC drugs. (4) Offering special course to medical, pharmaceutical, and nursing students, which emphasizes the specific behavior of rational PPI use. All those methods might facilitate the rational use of PPI.

## Supplementary information

**Additional file 1.** Questionnaire.

## Data Availability

The datasets generated and analyzed during the current study are not publicly available due the large amount of data analyzed, but they are available from the corresponding author on reasonable request.

## Abbreviations

GERD: Gastroesophageal reflux disease
NSAID: Nonsteroidal anti-inflammatory drug
OTC: Over-the-counter;
PPI: Proton pump inhibitor
SUP: Stress ulcer prophylaxis
