# Supplementary material for: Awareness, attitude and behavior regarding proton pump inhibitor among medical staff in the Southwest of China
Source: BMC Health Serv Res. 2019 Nov 21;19:880. doi: 10.1186/s12913-019-4725-6 (PMC6873501; doi:10.1186/s12913-019-4725-6)
Supplement: Supplementary file 1 — Additional file 1. Questionnaire. [file 12913_2019_4725_MOESM1_ESM.docx]

**Awareness regarding proton pump inhibitor among medical staff**

**Section A Demographics**

| No | Question | Answer |
| --- | --- | --- |
| 1 | Gender | □Male □Female |
| 2 | Age | □18~30 □31~40 □41~50 □51~60 |
| 3 | Education | □High school □Associate degree □Baccalaureate □Master degree □Doctorate |
| 4 | Professional title | □Primary □Secondary □Senior |
| 5 | Hospital nature | □Public □Private |
| 6 | Hospital type | □Comprehensive □Specialized |
| 7 | Hospital grade | □Grade-one □Secondary □Tertiary |

**Section B The awareness of respondents regarding PPI knowledge**

| No | Question | Answer |
| --- | --- | --- |
| 1 | Is PPI inactive prodrug? | □Yes □No |
| 2 | Do PPIs include omeprazole, pantoprazole, lansoprazole, rabeprazole, esomeprazole, etc? | □Yes □No |
| 3 | Do PPI cure acid-related diseases by suppressing hydrochloric acid secretion? | □Yes □No |
| 4 | Can PPI be used to prevent stress ulcer? | □Yes □No |
| 5 | Can PPI be used to treat acute pancreatitis? | □Yes □No |
| 6 | Does omeprazole have the largest individual difference compared with other PPIs? | □Yes □No |
| 7 | Does omeprazole have the largest interaction compared with other PPIs? | □Yes □No |
| 8 | Does esomeprazole have the longest acid inhibition time compared with other PPIs? | □Yes □No |
| 9 | Should omeprazole be selected for pediatric patients? | □Yes □No |
| 10 | Should rabeprazole be selected for pregnant patients? | □Yes □No |
| 11 | Do you think the more or new PPI will produce better and safer effect? | □Yes □No |
| 12 | Is PPI usually available as enteric-coated capsules or tablets ? | □Yes □No |
| 13 | Should PPI usually be taken at breakfast? | □Yes □No |
| 14 | Should PPI be taken after meal? | □Yes □No |
| 15 | Should PPI be swallowed as whole piece? | □Yes □No |
| 16 | Is it advisable to increase the dose frequency rather than a single dose to improve effect? | □Yes □No |
| 17 | Should patients take PPI for only 7 days in the Helicobacter pylori eradication therapy? | □Yes □No |
| 18 | Does PPI treatment of gastric ulcer take 2 weeks to 4 weeks? | □Yes □No |
| 19 | Is duration of PPI prophylaxis until no high risk factors, or able to tolerate enteral feeding? | □Yes □No |
| 20 | Do you think long-term use of PPI may cause adverse reactions such as osteoporosis, pneumonia, etc? | □Yes □No |

**Section C The attitude of respondents regarding PPI use**

| No | Question | Answer |
| --- | --- | --- |
| 1 | Overuse of PPI is common at present in China. | □Completely agree □Almost agree □Indifferent □Almost disagree □Completely disagree |
| 2 | The main cause of PPI overuse is doctors’ or patients’ abuse of PPI. | □Completely agree □Almost agree □Indifferent □Almost disagree □Completely disagree |
| 3 | The main purpose of PPI overuse is SUP. | □Completely agree □Almost agree □Indifferent □Almost disagree □Completely disagree |
| 4 | Overuse of PPI will cause an increase in adverse drug reaction and medical cost. | □Completely agree □Almost agree □Indifferent □Almost disagree □Completely disagree |
| 5 | Necessary to carry out large scale education on rational use of PPI for medical staff and the public. | □Completely agree □Almost agree □Indifferent □Almost disagree □Completely disagree |
| 6 | Necessary to strengthen the management of community pharmacy. | □Completely agree □Almost agree □Indifferent □Almost disagree □Completely disagree |

**Section D The behavior of respondents toward PPI use**

| No | Question | Answer |
| --- | --- | --- |
| 1 | Have you used proton pump inhibitors in the past one year? | □Yes □No |
| If you have used PPI, then the next 6 questions would be answered. | | |
| 2 | What's the name of the proton pump inhibitor you used? | □Omeprazole□Pantoprazole□Lansoprazole  □Rabeprazole □Esomeprazole |
| 3 | Use PPI when abdominal pain | □Always □Often □Sometimes  □Seldom □Never |
| 4 | Use PPI when ventosity | □Always □Often □Sometimes  □Seldom □Never |
| 5 | Use PPI when nausea | □Always □Often □Sometimes  □Seldom □Never |
| 6 | Use PPI when vomiting | □Always □Often □Sometimes  □Seldom □Never |
| 7 | Use PPI when acid reflux | □Always □Often □Sometimes  □Seldom □Never |
